# Nonvolatile Resistive Switching Memory Utilizing Cobalt Embedded in Gelatin

**DOI:** 10.3390/ma11010032

**Published:** 2017-12-26

**Authors:** Cheng-Jung Lee, Yu-Chi Chang, Li-Wen Wang, Yeong-Her Wang

**Affiliations:** Institute of Microelectronics, Department of Electrical Engineering, National Cheng-Kung University, Tainan 701, Taiwan; s.w.l.f.dd@gmail.com (C.-J.L.); s0964348@hotmail.com (Y.-C.C.); jk220052@gmail.com (L.-W.W.)

**Keywords:** cobalt, filament, gelatin, resistive memory

## Abstract

This study investigates the preparation and electrical properties of Al/cobalt-embedded gelatin (CoG)/ indium tin oxide (ITO) resistive switching memories. Co. elements can be uniformly distributed in gelatin without a conventional dispersion procedure, as confirmed through energy dispersive X-ray analyzer and X-ray photoelectron spectroscopy observations. With an appropriate Co. concentration, Co. ions can assist the formation of an interfacial AlO*_x_* layer and improve the memory properties. High ON/OFF ratio, good retention capability, and good endurance switching cycles are demonstrated with 1 M Co. concentration, in contrast to 0.5 M and 2 M memory devices. This result can be attributed to the suitable thickness of the interfacial AlO*_x_* layer, which acts as an oxygen reservoir and stores and releases oxygen during switching. The Co. element in a solution-processed gelatin matrix has high potential for bio-electronic applications.

## 1. Introduction

Gelatin has excellent film formation properties and biodegradability, as well as superior solubility in water. In addition, gelatin memory devices possess a good ON/OFF ratio in the atmospheric environment for 90 days [1]. However, gelatin memory devices suffer from poor switching cycle endurance.

Most organic memory devices use metallic nanoparticles blended into an organic host to improve their memory properties and operational stability [2,3,4,5]. Organic nanocomposites, by dispersing metal nanoparticles into organic matrixes, possess greatly enhanced mechanical, optical, and electrical properties. However, metal nanoparticle dispersion in organic matrixes can weaken the repeatability of devices due to strong inter-particle interactions and weak polymer nanoparticle interfacial interaction (nanoparticle agglomeration). Moreover, controlling the size of metal nanoparticles is difficult [1]. 

In this study, cobalt-embedded gelatin (CoG) was used as an organic insulator layer, which is a one-component system that considerably simplifies the device structure and fabrication process. Based on the Al/CoG/ITO device structure, three Co.:gelatin mole ratios (2:1, 1:1, and 0.5:1) were adopted in the experiments. Device performance improvements were summarized and analyzed. With appropriate Co. concentration, the redox reaction of Co. can assist in the formation of the interfacial AlO*_x_* layer and improve the memory properties. Compared with CoG 0.5 M and 2 M memory devices, CoG 1 M memory devices exhibit good device performance, being forming-free, with a sufficient ON/OFF ratio (larger than 10^5^), relatively good switching cycles, and extrapolated retention properties exceeding 10 years. According to the electrical properties and material analyses, the sufficient thickness of the interfacial AlO*_x_* layer and the smooth dielectric thin film is related to an appropriate Co. concentration in the gelatin matrix, which further enhances switching cycle endurance and retention capability.

## 2. Results

**Figure 1:**
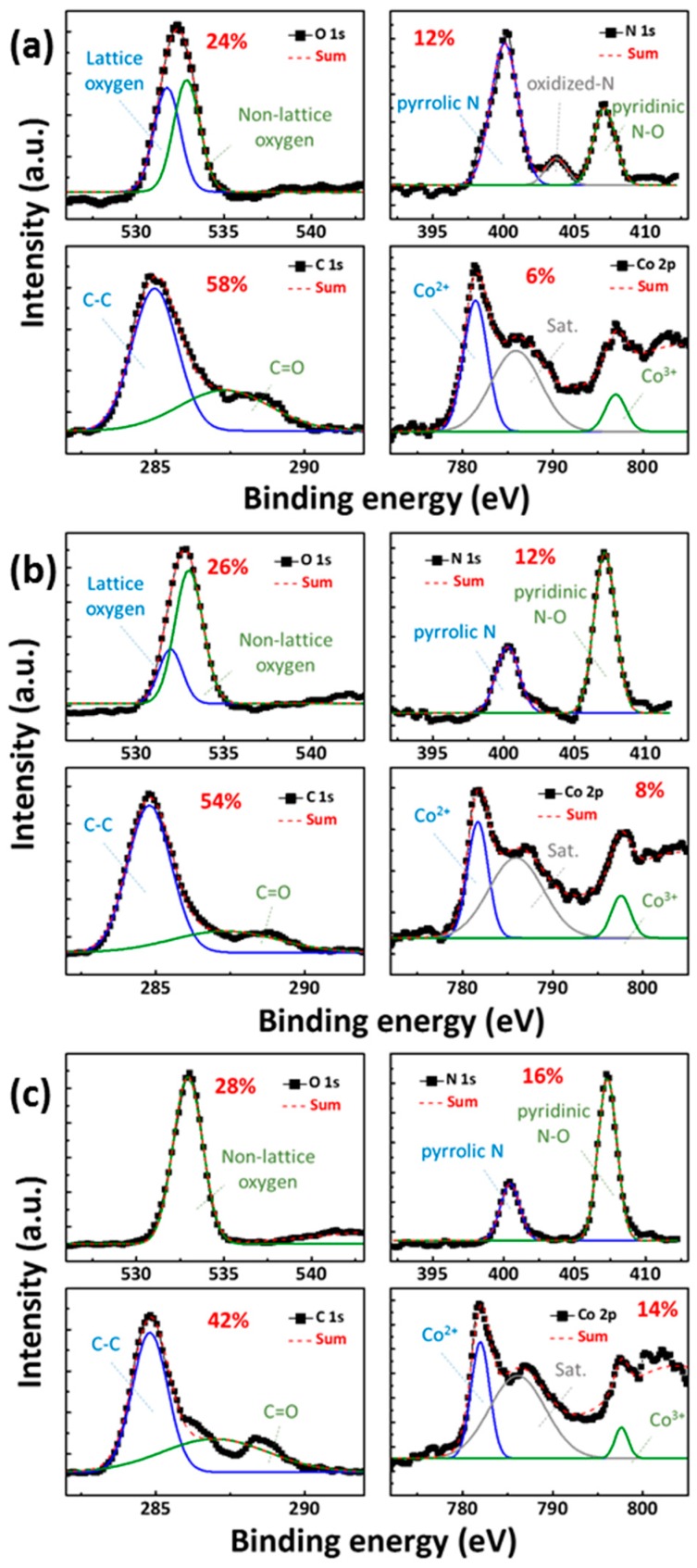
X-ray photoelectron spectroscopy (XPS) survey spectra of cobalt embedded gelatin (CoG) (**a**) 0.5, (**b**) 1, and (**c**) 2 M thin films.

Figure 1a–c shows the X-ray photoelectron spectroscopy (XPS) patterns obtained after 90 s of Ar+ ion sputtering, thereby representing the bulk layer of CoG 0.5, 1, and 2 M thin films, respectively. XPS quantitative analyses revealed that the atomic percentages of Co. were 6%, 8%, and 14% for CoG 0.5, 1, and 2 M thin films, respectively. The Co. concentration tendency in the energy-dispersive X-ray spectroscopy (EDS) element mapping is consistent with the XPS quantitative analyses.

The fitting of high-resolution N 1s XPS peaks shows the presence of pyrrolic-N, oxidized-N, and pyridinic-N [6], with a relatively high content of pyridinic-N and pyrrolic-N in the CoG thin film. The simulated characteristics of Co. 2p peaks were able to be divided into two peaks at around 781.5 eV and 797.4 eV, which confirmed the existence of Co^2+^ and Co^3+^ in the CoG thin films by XPS analysis [7,8]. The O 1s peaks were able to be divided into two peaks at 530 eV and 532 eV, which were assigned to lattice and non-lattice oxygen, respectively. The C 1s peaks were able to be divided into two peaks at 284 eV and 288 eV, which were assigned to epoxyl C–O and carboxyl O–C=O, respectively.

**Figure 2:**
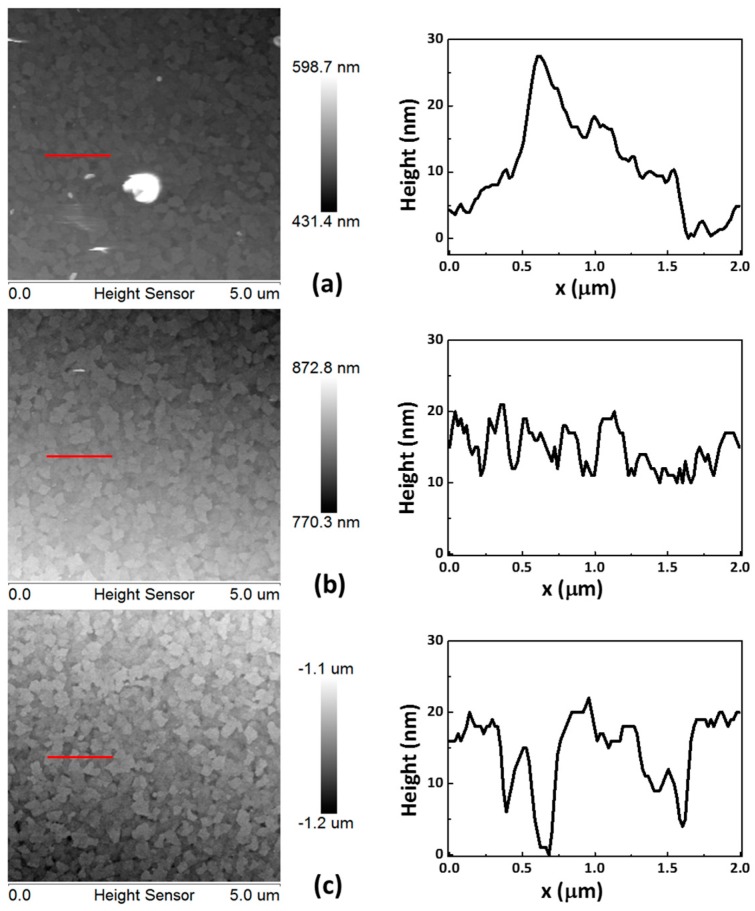
Atomic force microscope (AFM) image of the CoG (**a**) 0.5, (**b**) 1, and (**c**) 2 M thin films. The height profiles shown are taken along the red lines.

Figure 2a–c illustrates the surface topographies of the CoG 0.5, 1, and 2 M samples, respectively. The roughness values of the CoG 0.5, 1, and 2 M thin films are 5.1, 2.8, and 3.6 nm, respectively. These parameters confirm that CoG 1 M has the smoothest surface.

**Figure 3:**
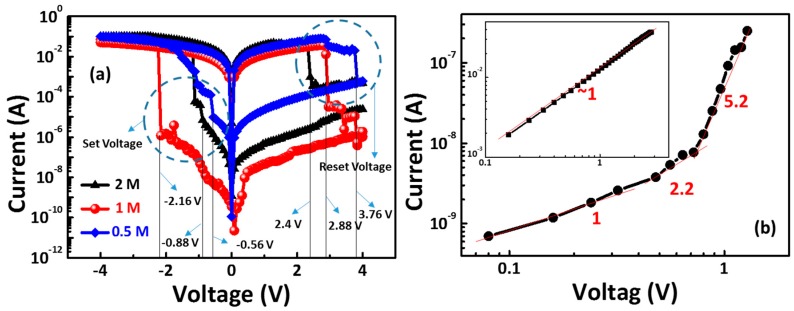
(**a**) Typical *I*–*V* curves of the Al/CoG (concentrations of Co. = 0.5, 1, and 2 M)/ITO structures. (**b**) I–V fitting of the high-resistance state (HRS) and low-resistance state (LRS) for the Al/CoG/ITO structure.

Figure 3a depicts the *I*–*V* characteristics of the Al/CoG 0.5 M/ITO, Al/CoG 1 M/ITO, and Al/CoG 2 M/ITO structures. Bipolar resistive switching behaviors are clearly observable. Similar to gelatin, the bipolar resistive switching behavior does not require an electroforming process. The forming voltage is usually far greater than the set voltage. In the CoG memory device case, the first and second *I*–*V* curves of the CoG 1 M memory device show nearly the same set voltage of around 1.3 V. Thus, a high-voltage forming process was avoided in the CoG memory device. Voltage series of 3 to −3 V, 4 to −4 V, and 5 to −5 V were used to operate the memory devices in this study. However, the voltage series of 3 V to −3V was insufficient to switch the device from a high-resistance state (HRS) to a low-resistance state (LRS). The voltage series of 5 V to −5 V has similar *I*–*V* characteristics with the voltage series of 4 V to −4 V. The switches can be operated with a smaller bias voltage of 4 V to −4 V, which may be preferable for memory devices with lower power consumption. Thus, the voltage was applied to the top Al electrode in a sequence of +4 V → 0 V → −4 V → 0 V → +4 V. The high ON/OFF ratio of over 10^5^ can be achieved using a Co. concentration of 1 M. 

Figure 3b shows how the *I*–*V* fittings resulted in low-resistance state (LRS) and high-resistance state (HRS), in order to better understand the switching mechanism of the CoG memory device. The space-charge-limited conduction (SCLC) model comprises three different conductive regions [9,10]: (1) a low-voltage region, which is the *I*–*V* curve with a slope of approximately 1 (*I* ∝ *V*) corresponding to the ohmic conduction mechanism; (2) a transition region where the slope increased to 2.2; and (3) considerable defects in the CoG 1 M thin film, such as non-lattice oxygen ions, as demonstrated in the XPS results. Defects in the thin film can form trap sites below the conduction band, where the injected charge carriers can be entrapped. When all the available traps are filled, the current density abruptly increases with an *I*–*V* slope larger than 2. In addition, the ln(I)–V^1/2^ and ln(I/V)–V^1/2^ plots of the *I*–*V* curve at the HRS were also investigated. However, the slope is not equal to 1 in both plots. Therefore, the HRS fitting result indicates that the SCLC is the dominant mechanism.

**Figure 4:**
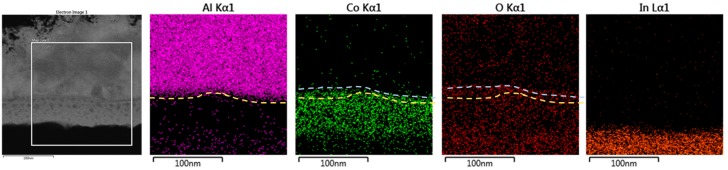
TEM cross-sectional energy-dispersive X-ray spectroscopy (EDS) mapping of the CoG 1 M memory devices.

Figure 4 shows the TEM cross-sectional EDS mapping of the CoG 1 M thin film. When a positive bias is applied to the Al electrode, Co. ions drift toward the ITO electrode due to the applied electric field between the Al and ITO electrodes via an ion conduction process in the CoG layer. When the Co. ions are near the ITO electrode, they are subjected to a reduction process wherein the ITO supplies electrons, as shown in Equation (1).

ne^−^ + Co^3+^ → Co.^(3−*n*)+.^ (1)

At the anode, the oxidation reaction may lead to the evolution of oxygen gas according to the following equation:

O_o_ → 2e^−^ + V_o_^2+^ + 1/2O_2,_ (2)

where V_o_^2+^ denotes oxygen vacancies with a double-positive charge with respect to the regular lattice, and O_O_ represents an oxygen ion. As an alternative to Equation (2), the metal electrode nearby may be oxidized [11]. The spontaneous oxidation of Al at the Al–CoG interface causes AlO*_x_* formation because Al is chemically active [12]. The interface between the Al and CoG thin film comprises Al, Co., and O.

**Figure 5:**
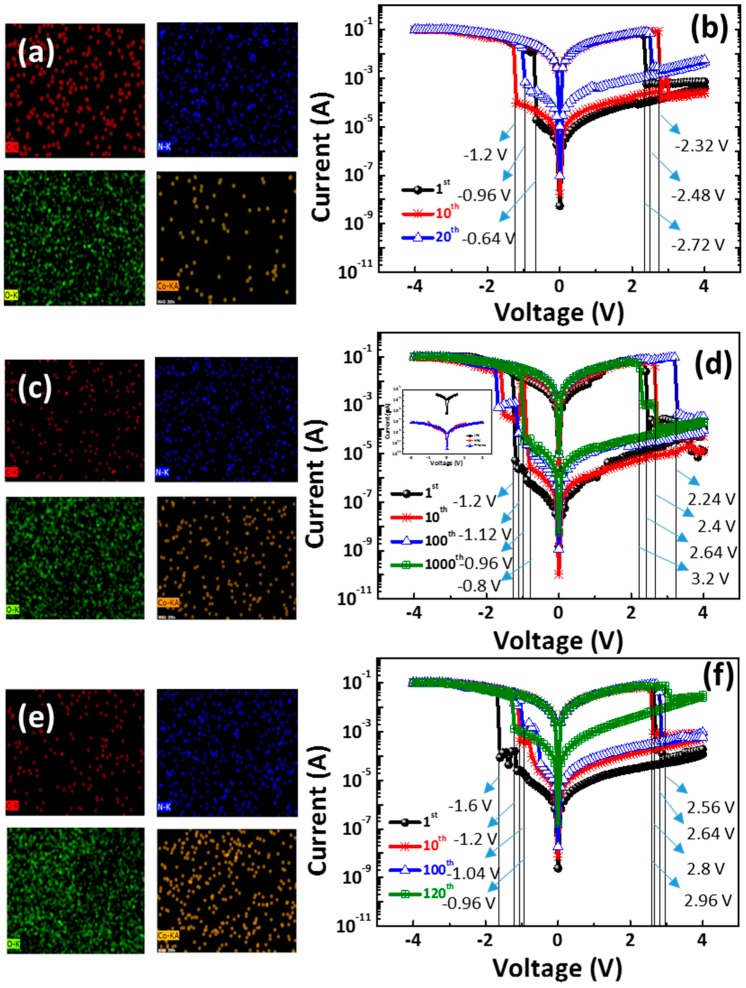
Here, EDS C (red), N (blue), O (green), and Co. (orange) element mapping images of CoG (**a**) 0.5, (**c**) 1, and (**e**) 2 M thin films are shown. The switching cycles of (**b**) Al/CoG (0.5 M)/ITO, (**d**) Al/CoG (1 M)/ITO, and (**f**) Al/CoG (2 M)/ITO structures can also be seen. The inset of (**d**) shows the low-field *I*–*V* characteristics corresponding to a typical pristine state (LRS and HRS).

Figure 5a,c,e shows the EDS of C (red), N (blue), O (green), and Co. (orange) element mapping images of the CoG 0.5, 1, and 2 M thin films. The chemical structure of the gelatin is Ala-Gly-Pro-Arg-Gly-Glu-4Hyp-Gly-Pro [13], which has a heteroatom (i.e., nitrogen or silicon) that is strongly coordinated with metal ions. However, the XPS analysis shows that the metallic Co. does not exist in the CoG thin film. Thus, an agglomeration of the Co. could be observed in the small region. However, Co. elements are uniformly distributed in most regions of the CoG thin film.

Figure 5b,d,f shows the switching *I*–*V* curves of the Al/CoG (0.5 M)/ITO, Al/CoG (1 M)/ITO, and Al/CoG (2 M)/ITO structures under the DC voltage sweep. Increasing the Co. concentration up to 1 M increases the switching cycle, but it decreases when the Co. concentration reaches 2 M. The Al/CoG (1 M)/ITO structure can maintain an ON/OFF ratio of over 10^3^ after continuous DC voltage switching cycles of 1000 times. The low-field *I*–*V* characteristics corresponding to a typical pristine state (LRS and HRS) are shown in the inset of Figure 5d. All clearly exhibit significant field-dependent conductivity. The conductivity of the pristine state is similar to that of the HRS. The initial conduction property of CoG is relatively insulated.

**Figure 6:**
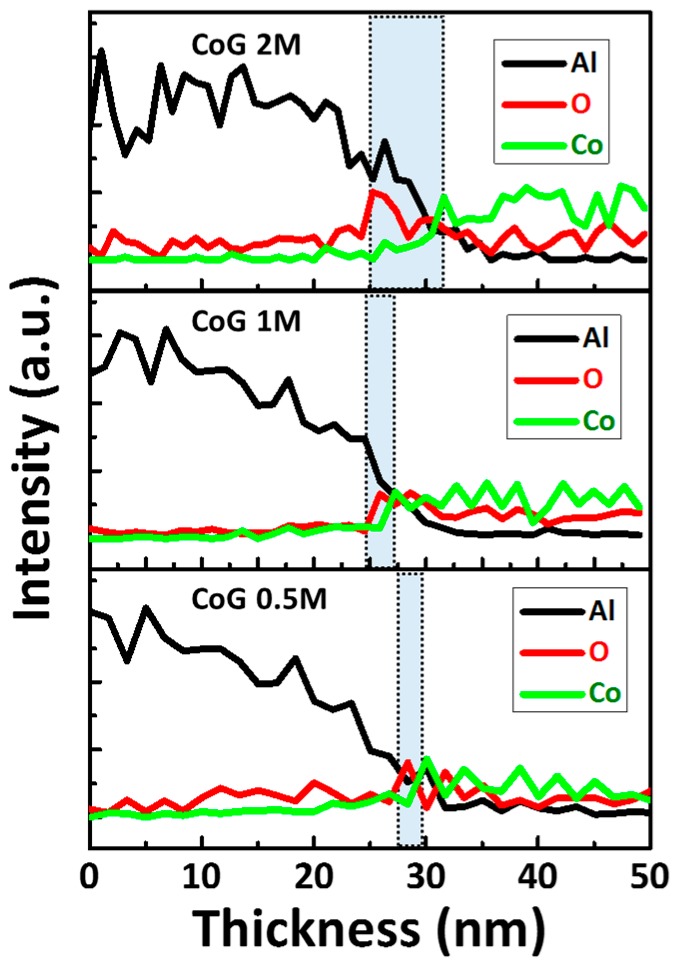
TEM line-scan profiles of the CoG 0.5 M, 1 M, and 2 M memory devices.

The thicknesses of native Al oxide were determined using TEM line-scan profiles. Figure 6 shows the TEM line-scan profiles of the CoG 0.5 M, 1 M, and 2 M memory devices. The native Al oxide layer measures around 5, 2, and ~1 nm for the CoG 2 M, 1 M, and 0.5 M memory devices, respectively.

**Figure 7:**
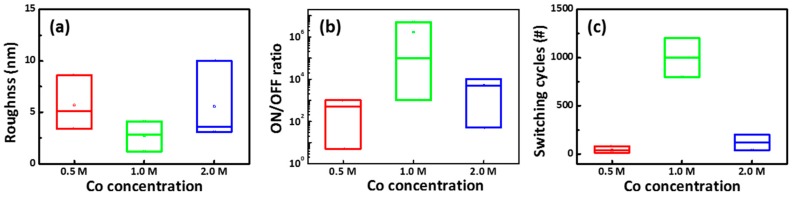
Relationship among the (**a**) R_rms_; (**b**) ON/OFF ratio; (**c**) switching cycles, and Co. concentrations in the Al/CoG/ITO (read at 0.5 V).

Figure 7a shows the relationship of the R_rms_ as a function of Co. concentration in the Al/CoG/ITO structure. The average R_rms_ values were counted by measuring six points in a 1.5 cm × 1.5 cm sample for each Co. concentration. As shown in Figure 7b,c, the statistical results for ON/OFF ratio and switching cycles of CoG 0.5, 1, and 2 M memory devices were randomly obtained from 20 out of 25 devices (80% in yield) in three fabricated substrates. Among these Co. concentrations, the smoothest surface, highest ON/OFF ratio, and best switching cycles occurred at 1 M Co. concentration. This result can be attributed to smooth roughness, which facilitates the stable resistive switching of the Resistive random-access memory (RRAM) device [14].

**Figure 8:**
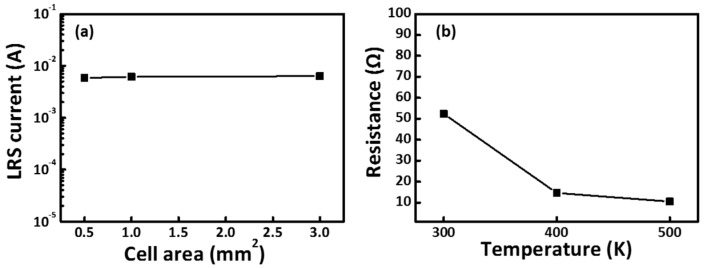
(**a**) LRS current versus cell area; and (**b**) electrical transport thermally activated for the set states of the CoG 1 M memory device.

Figure 8a plots the scaling trend of the LRS current versus the cell area of the CoG 1 M memory device. The LRS current is mainly a filamentary conduction current, and the LRS only has a slight dependence on the cell area [15]. The independent LRS current with cell area confirms the filamentary nature of resistive switching in the CoG memory device. Temperature dependence of electrical transport can be used to gain insight into the nature of these conductive filaments. Figure 8b shows the temperature dependence of the LRS current in the CoG memory device. The thermally activated transport behavior excludes the possibility that the LRS comprises metallic filaments. CoG 1 M memory devices have improved endurance cycles. This property can be attributed to partial dissociation of conductive filaments in the CoG layer; by contrast, conductive filaments remain stable in the interfacial AlO*_x_* layer during subsequent switching cycles. The existence of the interfacial AlO*_x_* layer could considerably improve the device properties. The presence of the AlO*_x_* layer at the interface between CoG thin film and Al electrode correlates with the additional DC switching cycles observed in the Al/CoG 1 M/ITO cell. Intermediate oxide layers influenced their conduction behavior [16,17,18]. The suitable thickness of the interface AlO*_x_* layer acts as a diffusion barrier that prevents the infusion of oxygen into the atmosphere, similarly to an oxygen reservoir that stores and releases oxygen during switching. 

**Figure 9:**
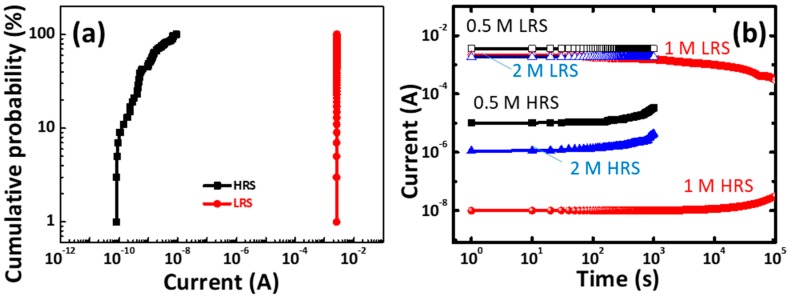
(**a**) Statistical distributions of current values of Al/CoG (1 M)/ITO structure; (**b**) Retention characteristics of the CoG (0.5, 1, and 2 M) memory devices.

Figure 9a presents the statistical distribution parameters of the gelatin memory device The LRS and the HRS currents are measured at 0.1 V. Although the HRS was relatively broad, the LRS was well separated from the HRS, thereby allowing the two states to be distinguished. Figure 9b presents the retention capability of CoG 0.5, 1, and 2 M memory devices measured at room temperature and obtained at a voltage of 0.1 V. The ON/OFF ratio of the CoG memory device (1 M) remains higher than 10^5^. The smooth roughness may facilitate stable resistive switching [19,20], and an improved retention capability is also achieved.

## 3. Materials and Methods

CoG thin films were fabricated on ITO/glass substrates by solution process. ITO/glass substrates (Aim Core Technology, Hsinchu, Taiwan) were cut to 2.0 cm × 1.5 cm and cleaned using acetone, methanol, and de-ionized (DI) water in an ultrasonic bath. Cobalt nitrate hexahydrate (Co.(NO_3_)_2_∙6H_2_O) and gelatin powder were added to DI water and stirred until the powders were completely dissolved. Then, the CoG solutions were spun on the cleaned ITO/glass substrates and baked at 80 °C for 10 min. The top electrode area of Al measured 3 mm^2^. The molar ratios of Co. and gelatin were fixed to 0.5:1, 1:1, and 2:1, and the Al/CoG 0.5 M/ITO, Al/CoG 1 M/ITO, and Al/CoG 2 M/ITO structures were fabricated. 

Atomic force microscope (AFM) analysis (Dimension ICON with NanoScope V controller, Bruker, Billerica, MA, USA) was used to characterize the surface morphology and roughness of the obtained CoG/ITO samples. Scanning electron microscope (SEM) analysis was carried out using Hitachi SU8000 systems. The elements of the samples before measurement were mapped by EDX analysis (Bruker, Ettlingen, Germany). The electrical characteristics were measured by Agilent B1500 semiconductor characterization system (Agilent, Santa Clara, CA, USA). 

## 4. Conclusions

In summary, a memory device based on simple solution-processed CoG thin film has been demonstrated. The uniform distribution of Co. in the CoG thin film can be observed by the EDX analyzer. The thicknesses of interfacial AlO*_x_* layer are suggested to increase with Co. concentration due to the assistance of the Co. redox reaction in the interfacial AlO*_x_* layer formation. The solution-processed Al/CoG 1 M/ITO structure exhibits excellent device performance, being forming-free, with a sufficient ON/OFF ratio of over 10^5^ and extrapolated retention properties exceeding 10 years. In addition, increasing the Co. concentration up to 1 M increases the switching cycle, although it decreases when the Co. concentration reaches 2 M. The CoG 2 M memory device always fails at the LRS state, which means that the oxygen vacancies cannot obtain sufficient oxygen ions to rupture the filament paths. With Co. concentration of 1 M, the suitable thickness of the AlO*_x_* interface layer acts as an oxygen reservoir for storing and releasing oxygen during switching, which further improves the switching cycle endurance.
